# Selectivity of Monaural Synaptic Inputs Underlying Binaural Auditory Information Integration in the Central Nucleus of Inferior Colliculus

**DOI:** 10.3389/fncel.2018.00303

**Published:** 2018-10-04

**Authors:** Jinxing Wei, Wen Zhong, Cuiyu Xiao, Yun Liu, Changbao Song, Zhongju Xiao

**Affiliations:** Key Laboratory of Mental Health of the Ministry of Education, Key Laboratory of Psychiatric Disorders of Guangdong Province, Department of Physiology, School of Basic Medical Sciences, Southern Medical University, Guangzhou, China

**Keywords:** binaural acoustic response, excitatory postsynaptic current, inhibitory postsynaptic current, monaural selectivity, the central nucleus of inferior colliculus

## Abstract

Neurons in the central nucleus of the inferior colliculus (ICC) receive ascending inputs from the ipsilateral and contralateral auditory pathway. However, the contributions of excitatory or inhibitory synaptic inputs evoked by ipsilateral and contralateral stimuli to auditory responses of ICC neurons remain unclear. Using *in vivo* whole-cell voltage-clamp recordings, we investigated excitatory and inhibitory synaptic currents in neurons of the ICC in response to binaural stimulation by performing an intensity-intensity scan. To systematically analyze the contribution of the ipsilateral and contralateral ear, the sound intensity was randomly delivered to each side from 0 dB sound pressure level (SPL) to 70 dB SPL. Although the synaptic responses were dominated by contralateral inputs at weak sound intensities, they could be increased (or decreased) by additional ipsilateral stimulation at higher intensities. Interestingly, the synaptic responses to contralateral acoustic inputs were not linearly superimposed with the ipsilateral ones. By contrast, the responses showed either a contralateral or ipsilateral profile, depending on which one was more dominant. This change occurred at a certain intensity “switch” point. Thus, the binaural auditory responses of the ICC neurons were not simply mediated by the summation of the inputs evoked by ipsilateral and contralateral stimulations. This suggested that the ICC might inherit the acoustic information integrated at the brainstem, causing the selectivity of monaural excitation and inhibition to underlie the neuronal binaural acoustic response.

## Introduction

The central nucleus of the inferior colliculus (ICC) is a major processing and integrating center for acoustic information in the neuronal ascending auditory pathway (Winer and Schreiner, 2005). Anatomical and physiological studies suggest that most ICC neurons receive monaural (Greene et al., 2010; Malone and Schreiner, 2010; Young, 2010) and binaural (Adams and Mugnaini, 1984; Winer et al., 1995; Oliver, 2000; Oliver et al., 2003; Loftus et al., 2004; Ito and Oliver, 2010; Malmierca and Hackett, 2010) projections from lower auditory nuclei. Whether the ICC is a relay for the ipsilateral and contralateral contributions and whether there are any other synaptic mechanisms involved remains unclear.

Previous studies mostly focused on the binaural auditory response to the interaural level differences (ILDs) of the stimulation. Neuronal responses to different ILDs are considered to be evidence of the integration of binaural information (Davis et al., 1999; Park et al., 2004). Ono and Oliver (2014) have revealed that the acoustic-evoked excitatory and inhibitory inputs to the inferior colliculus (IC) neurons are balanced and encoded the ILDs in a complicated way by showing different response amplitudes or charges. Complex integrations are suggested to exist within the IC based on the unchanging, facilitating, or inhibiting effects of ipsilateral stimulation on the binaural acoustic response. The excitatory/inhibitory (EI) neurons (excited by a stimulus applied to one ear while suppressed by stimulation to the other) in the IC could inherit the excitatory and inhibitory properties integrated in the lower auditory nuclei, such as the lateral superior olive (LSO) and the dorsal nucleus of lateral lemniscus (DNLL; Li and Pollak, 2013). Additionally, Xiong et al. (2013) demonstrated that binaural interactions of excitatory inputs on the ICC neurons could be shaped within other auditory nuclei in the brainstem, while the neuronal circuit mediating the inhibitory inputs to the ICC is complex. However, the contribution of the monaural inputs to the ICC neuronal binaural response is not fully understood, nor is it understood whether the excitatory/inhibitory inputs evoked by contralateral and ipsilateral stimulation are integrated in the ICC.

**Figure 1:**
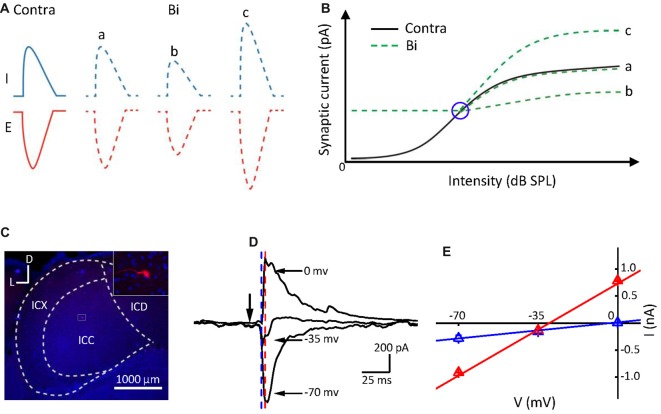
Postsynaptic currents evoked by binaural acoustic stimuli in central nucleus of the inferior colliculus (ICC) neuron. **(A)** Three different binaural synaptic responses were observed. E, excitatory; I, inhibitory. **(B)** The black line represents contralateral intensities that varied from low to high (from left to right). Green lines indicate three potential forms of binaural synaptic response when the ipsilateral input was fixed at a higher intensity and contralateral intensities were varied from low to high (from left to right). The blue circle indicates the “switch” point. The *x*- and *y*-axes represent the stimulation intensity and synaptic response amplitude, respectively. **(C)** Confocal image of the IC area and the biocytin-labeled neuron in the ICC. D, dorsal; L, lateral. **(D)** Average synaptic currents (10 repeats) of an example cell evoked by a characteristic frequency (CF) tone at 70 dB sound pressure level (SPL), recorded at different holding potentials from the same neuron shown in **(C)**. Black arrowhead indicates the onset of tone stimuli. **(E)** Current-voltage (I–V) relationship plotted for the same cell in **(C,D)**. The current value was averaged within a 1 ms window at 11 ms post tone onset (just before the onset of the inhibitory current, marked by blue dashed line) and at 14 ms post tone onset (around the peak of inhibitory current, marked by red dashed line), respectively. Bar = SE. Note that the reversal potential measured for the current values at the time point marked by the blue dashed line is approximately 0 mV.

We speculate that the monaural contribution on the neuronal binaural response should depend on their relative strength. Ipsilateral stimulation modified the ICC neuronal synaptic (excitatory and inhibitory) responses evoked by contralateral stimulation (Ono and Oliver, 2014), indicated in Figure 1A. If the contralateral stimulation intensity increases (from left to right, Figure 1B, the *black line*) while the ipsilateral one is constant, the neuronal binaural responses would exhibit an intensity response curve (Figure 1B, the *green lines*). The contralateral responses would be unchanged: (a); inhibited (b); or facilitated (c) by the ipsilateral stimulation that induced relatively low responses (Figure 1B, the right portion beyond the *blue circle*). However, when the ipsilateral responses were larger than the contralateral responses (Figure 1B, the left portion beyond the *blue circle*), the binaural responses should be consistent with the ipsilateral responses. If this is the case, the monaural selectivity of excitation and inhibition would be a mechanism underlying the integration of the binaural information. The ICC binaural acoustic response should be mediated simply by the summation of the synaptic inputs induced by ipsilateral and contralateral stimulation if this information was integrated within the ICC itself. If the binaural acoustic response was not mediated simply by summation, then this suggests that the binaural information was integrated before reaching the ICC.

To verify the above hypothesis, we investigated the excitation/inhibition of the ICC neurons to binaural stimulation with that of contralateral or ipsilateral stimulation by using *in vivo* whole-cell voltage-clamp recordings and performing an intensity-intensity scan to the animal (Kyweriga et al., 2014). By analyzing the relationship between the binaural and monaural synaptic inputs, we found that the ipsilaterally presented sound could have no effect on, suppress, or enhance the binaural response. However, no matter how ipsilateral inputs affected the binaural responses, the only ipsilateral inputs or the contralateral inputs modified by the ipsilateral ones were selected depending on their relative strength. A cutoff (such as shown in Figure 1B, the *blue circle*) between the contralateral and ipsilateral dominations was defined as a “switch” point in this study. Our results indicated that ICC neurons could inherit the monaural acoustic inputs after they have been modified/integrated at the brainstem.

## Materials and Methods

### General

Fifteen female C57BL/6 mice aged 4–6 weeks and weighing 14–20 g (Experimental Animal Center of Southern Medical University, Guangzhou, China) were used in this study. Our experimental protocols were approved by the Animal Care and Use Committee of Southern Medical University, Guangzhou, China. All efforts were made to minimize the number of animals used and their suffering. The methods for animal surgery preparation and acoustic stimulation in the present study were based on those described in our previous work (Wang et al., 2013; Xiong et al., 2013; Huang et al., 2015).

### Animal Preparation

Before the experiments, the mice underwent surgery with sodium pentobarbital (60–70 mg/kg, i.p.) and atropine sulfate (0.25 mg/kg, s.c.), which was administered to each mouse for anesthesia and for the inhibition of respiratory secretions, respectively. During the surgical preparation, the pedal withdrawal reflex of the animal was checked, and anesthesia was maintained by supplemental doses of sodium pentobarbital (13 mg/kg). The animal’s body temperature was continuously monitored and maintained at 37.5°C using a heating pad with a feedback controller. The animal’s head was fixed to a stereotaxic apparatus using ear bars, and the scalp was removed. A reference electrode was placed in the prefrontal cortex. Later, a 1.5 cm nail was fixed to the skull surface with dental cement. After that, the skull over the ICC (according to the atlas for the mouse brain: −5.2 mm from Bregma, 1 mm lateral to the midline) was opened (0.5 × 0.5 mm^2^) without the removal of the dura. The exposed brain was covered with Vaseline to prevent desiccation. During the surgery, lidocaine hydrochloride was used as a local anesthetic. After surgery, antibiotic ointment was applied to the surgical wound once a day. The mouse was returned to a cage with food and water to recover for at least 7 days.

### *In vivo* Whole-Cell Recordings

After atropine sulfate (0.25 mg/kg) was injected subcutaneously to reduce tracheal mucous secretion, and mice were anesthetized with urethane (1.2 g/kg, i.p.). Subsequently, the nail was inserted into a small metal rod and fixed by screws to immobilize the mouse’s head, which was tightly connected to an anti-vibration table (TMC, Peabody, MA, USA) in a soundproof room (temperature maintained at 24–26°C). After the Vaseline and dura were removed, a glass micropipette (tip diameter: approximately 1.5 μm, impedance: 4–7 MΩ) was inserted into the ICC region vertically with a micromanipulator (Siskiyou Inc, Grants Pass, OR, USA) up to a depth of 500–1,400 μm below the brain surface. For *in vivo* whole-cell recordings (Wu et al., 2008; Wang et al., 2017), the electrode pipettes were filled with a solution containing the sodium channel blocker QX-314 to block the firing of action potentials. The intrapipette solution contained (in mM) the following: 125 Cs-gluconate, 5 TEA-Cl, 4 MgATP, 0.3 GTP, 8 phosphocreatine, 10 HEPES, 10 EGTA, 2 CsCl, 1 QX-314 and 26 biocytin. The pH was adjusted to 7.25 using cesium hydroxide, and the osmotic pressure was approximately 295 mOsm. Recordings were made with an Axopatch 700B amplifier (Axon, Instruments/Molecular Devices, Sunnyvale, CA, USA). When a giga-ohm seal was formed between the glass pipette electrode and a neuron, suction was applied to the pipette for whole-cell recordings. For voltage-clamp recordings, the whole-cell capacitance and pipette capacitance were completely compensated, and the initial series resistance (20–40 MΩ) was compensated by 50%–60% to achieve an effective series resistance of 10–20 MΩ. Signals were filtered at 5 kHz and sampled at 10 kHz. Only neurons with resting membrane potentials lower than −55 mV and a stable series resistance were used for further analysis of whole-cell recordings. To obtain tone-evoked synaptic responses, the neurons were clamped at −70 mV and 0 mV, which were around the reversal potentials of inhibitory and excitatory currents, respectively, as described in previous studies (Tan et al., 2004; Wu et al., 2006).

### Acoustic Stimulation

The acoustic stimuli were generated using a Tucker-Davis Technologies System 3 (TDT 3, Tucker-Davis Technologies, Alachua, FL, USA). The sinusoidal signals were synthesized using a real time processor (RP2.1) and amplified by an electrostatic speaker driver (ED1). The intensities were controlled by a programmable attenuator 5 (PA5). Sounds were delivered by a closed loudspeaker (EC1, frequency range 0.1–100 kHz) through small metal tubes. The tip of the metal tube was inserted into the external auditory meatus. Similar to a previous study, the acoustic crosstalk gradually decreased with the stimulation frequency increase as measured by cochlear microphonic responses (Ono and Oliver, 2014). Additionally, by monitoring extracellular responses in the cochlear nucleus (CN), we found that the interaural attenuation was >60 dB sound pressure level (SPL) for frequencies >8 kHz, which was consistent with our previous work (Xiong et al., 2013). The loudspeaker was calibrated with 1/8 and 1/4 inch microphones and an amplifier (Brüel and Kjaer 4138, 4135 and 2610, Naerum, Denmark). The amplitude of pure tone bursts was expressed as the SPL (0 dB SPL, referred to 20 μPa). The parameters of the sound stimuli (frequency, intensity, duration and rise/fall time) were controlled by Brain Ware software (Tucker-Davis Technologies, Alachua, FL, USA) through a computer. Each acoustic stimulation sequence was repeated 10 times.

Neurons were first clamped at −70 mV to record the excitatory postsynaptic currents (EPSCs). Tone bursts (50 ms duration with 5 ms rise/fall time) of varying frequencies (2–64 kHz, at 0.1 octave interval) and intensities (0–70 dB SPL, in 10 dB SPL steps) were presented to the contralateral or ipsilateral ear separately or simultaneously in a randomized sequence. Subsequently, the neuron characteristic frequency (CF) was extracted. The CF was determined by finding the frequency that elicited the greatest response at the lowest intensity (Polley et al., 2007). To characterize binaural response properties, we used randomly interleaved tone bursts (50 ms duration, at CF), and it was presented simultaneously to each ear from 0 dB SPL to 70 dB SPL in 10 dB SPL steps (an array of 8 × 8 binaural stimuli). After we recorded the responses at −70 mV, we changed the holding potential to 0 mV to obtain inhibitory postsynaptic currents (IPSCs) and repeated the same sound protocol as above.

### Histology

After the experiments, mice were overdosed with sodium pentobarbital (100 mg/kg, i.p.) and perfused through an intracardiac catheter with 0.9% physiological saline, followed by 4% paraformaldehyde (PFA) in 0.1 M phosphate-buffered solution (PBS). The brain was removed from the skull and postfixed with 4% PFA for 24 h at 4°C. After 24 h immersion in 20% and 30% sucrose for cryoprotection, it was coronally sectioned at 40-μm thickness with a freezing microtome (Leica CM 1950, Nussloch, Germany). Free-floating sections were washed three times in PBS for 10 min each time. To increase the permeability of the antibody through the cell membrane, the sections were incubated with 0.3% Triton X-100 for 1–2 h. After the sections were rinsed three times for 10 min each in PBS, they were incubated with streptavidin-Cy3 (1:200, Molecular Probes, catalog no. 43-4315, Eugene, OR, USA) and bovine serum albumin (5%, Boster, AR0004, Wuhan, China) at room temperature for 3–4 h. Aluminum foil was used to shield the sections from light. The sections were then washed with distilled water and transferred to subbed slides. After drying, the sections were stained with 0.25 μg/mL 4’,6-diamidino-2-phenylindole (DAPI) to visualize neuronal cell nuclei. The slides were examined by a confocal microscope (Nikon, A1R, Japan). All the neurons recorded were stained with biocytin after the whole-cell recordings. To confirm that our recording sites specifically targeted the ICC, we inspected the glass pipette electrode tracks and the locations of labeled neurons according to the atlas for the mouse brain (Lein et al., 2007).

### Data Analysis and Statistics

The boundaries of synaptic response tonal receptive fields (TRFs) were defined with the custom-written software in MATLAB 2012b. The peak amplitude was measured from the baseline (which was calculated before the stimulus onset). The response threshold was the minimum stimulus intensity that induced a tone-evoked synaptic response in a cell. Synaptic response traces evoked by the same test stimuli were averaged. The onset latency was identified at the time point in the rising phase of the response waveform, where the amplitude exceeded the baseline current by two standard deviations (SDs). The values of peak amplitude, latency and half-width were calculated using the Microsoft Excel software (version 2010). All statistical analyses were performed using the SPSS statistical software (version 19). Paired *t*-tests were used for two-group comparisons. For three-group comparisons, one-way ANOVA (least significant difference (LSD) tests for multiple comparisons) was used to compare means. The *F*-test was used to evaluate the equivalence of variance. Significance was defined as *p* < 0.05. The data plotting was carried out using the Origin software (version 8). The results were presented as the mean ± SD, if not specified otherwise.

## Results

We recorded synaptic inputs from 25 ICC neurons in C57 BL/6 mice using whole-cell recording techniques in voltage-clamp mode. Both EPSCs and IPSCs were obtained on 23 neurons, while only IPSCs were obtained on the other two neurons. The mean depth of the recording sites was 924.2 ± 260.7 μm. The range of the CF was 8–32 kHz, and the mean of CF was 16.1 ± 6.0 kHz. The recording neurons were labeled with biocytin (Figure 1C), and data from neurons outside of the ICC were discarded. Most ICC neurons (20 out of the 25 cells) labeled in this study were identified as disc-shaped cells (Oliver et al., 1991), and the remaining cells were stellate neurons. An example of a well-clamped neuron is shown in Figures 1D,E. A linear current-voltage relationship (I–V curve) was observed for the recorded synaptic currents evoked by the CF tone at 70 dB SPL (CF = 16 kHz). The direction of the tone-evoked postsynaptic currents changed from negative to positive as the holding potential shifted from −70 mV to 0 mV. The CF tone at 70 dB SPL induced a small IPSC when the holding potential was at −35 mV and induced a pure IPSC when the holding potential was at 0 mV. Consistent with previous reports (Tan et al., 2004; Sun et al., 2010), in the auditory cortex, the tone induced an EPSC when the membrane potential was held at −70 mV and induced an IPSC when held at 0 mV.

**Figure 2:**
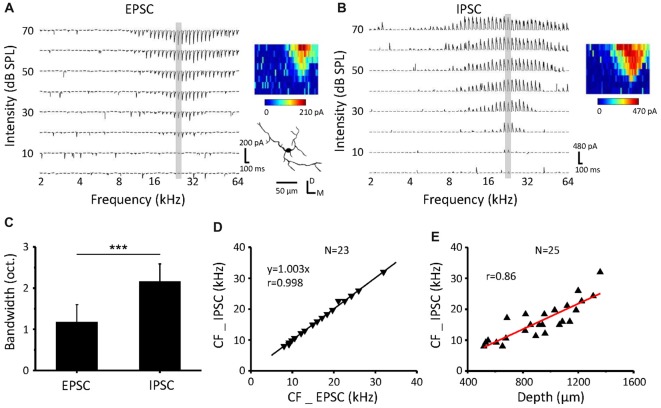
Inhibitory postsynaptic currents (IPSCs) have the same CF but wider BW than excitatory postsynaptic currents (EPSCs) as a response to contralateral stimulation **(A,B)**. An example neuron recorded from the ICC. Tonal receptive fields (TRFs) of synaptic currents evoked by pure tone stimuli at various frequencies and intensities were obtained, with the neuron clamped at −70 mV **(A)** and 0 mV **(B)**. The color maps on the right indicate the amplitudes of individual synaptic currents. **(C)** Average bandwidth synaptic TRFs at 60 dB SPL. ***P < 0.001, paired *t*-test. **(D)** Comparison of the CF between EPSCs and IPSCs for all the recorded cells. The dotted line is the best-fit linear regression line. **(E)** The relationship of recording depth and the CF.

Synaptic TRFs for an example neuron in the ICC are shown in Figures 2A,B. The bandwidth at 60 dB SPL (BW60) of excitatory synaptic inputs was significantly narrower than that of the inhibitory inputs (Figure 2C, paired *t*-test, *p* < 0.001, *n* = 23). The TRFs of excitatory and inhibitory inputs exhibit the same CF (Figure 2D) and so do the contralateral and ipsilateral TRFs, supporting our previous study (Xiong et al., 2013). The neuronal CFs were correlated with the recording depth (Figure 2E, *r* = 0.86) in accordance with a dorsal-to-ventral (low-to-high) gradient of CF (Willott, 1984; Stiebler and Ehret, 1985; Yu et al., 2005).

### A Linear Transformation of the Contralateral Response Into a Binaural Synaptic Response

IC neurons have been reported to respond to binaural acoustic stimulation (Irvine and Gago, 1990; Chhan et al., 2013; Xiong et al., 2013; Ono and Oliver, 2014; He et al., 2017). However, the differences in the synaptic inputs of ICC neurons between binaural stimulation and monaural stimulation are still unclear. We, first, set the binaural stimuli to the same intensity at both ears, mimicking the ILD for a sound source at the midline and, then, examined ICC neuron postsynaptic currents in response to the CF tone at different intensities presented to the mice contralaterally, ipsilaterally and binaurally in a random sequence using *in vivo* whole-cell recordings.

**Figure 3:**
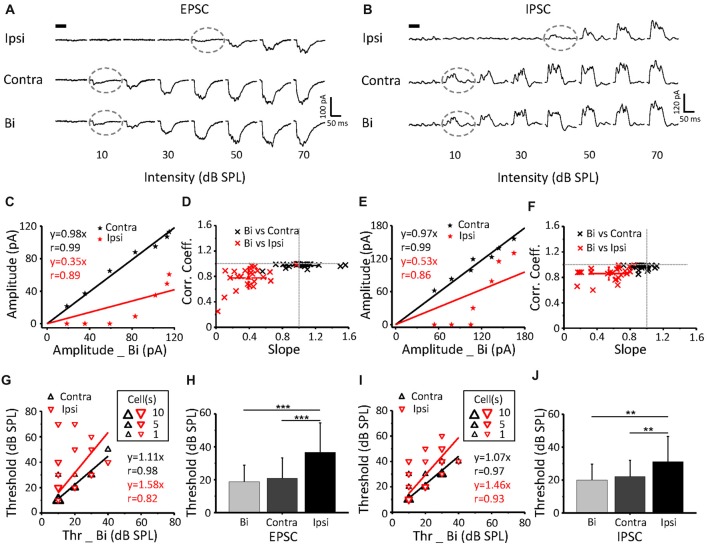
The relationship of binaural synaptic response against monaural responses when stimulus intensity was equal. **(A,B)** An example ICC neuron showed superimposed CF tone-evoked excitatory **(A)** and inhibitory **(B)** synaptic current responses under ipsilateral, contralateral and binaural stimulation. The black bar indicates the 50 ms long acoustic stimulus. The dashed lines indicate the threshold. **(C,E)** Binaural evoked excitatory **(C)** and inhibitory **(E)** synaptic responses vs. the corresponding contralateral (the black line) or ipsilateral (the red line) evoked response to the same tone plotted for the cell shown in **(A,B)**. **(D,F)** Distribution of the correlation coefficient vs. slope in the recorded population (excitatory **(E)** *n* = 23; inhibitory **(F)** *n* = 25). **(G,I)** Binaural synaptic response thresholds vs. the corresponding contralateral and ipsilateral thresholds. **(H,J)** Comparison of the thresholds of EPSCs **(H)** and IPSCs **(J)** among ipsilateral, contralateral and binaural stimulation. Contra-EPSC, 20.9 ± 12.4 dB SPL; ipsi-EPSC, 36.5 ± 18 dB SPL; and binaural-EPSC, 18.7 ± 10.1 dB SPL; *n* = 23; one-way ANOVA, *p* < 0.001; least significant difference (LSD) tests for multiple comparisons, *p* = 0.598 (contra-binaural). Contra-IPSC, 22.4 ± 10.9 dB SPL; ipsi-IPSC, 31.2 ± 15.4 dB SPL; and binaural-IPSC, 20 ± 9.6 dB SPL; *n* = 25; one-way ANOVA, *p* < 0.01; LSD tests for multiple comparisons, *p* = 0.556 (contra-binaural). **p < 0.01; ***p < 0.001. Data are expressed as the mean ± SEM.

Excitatory (Figure 3A) and inhibitory (Figure 3B) synaptic currents obtained from the same neuron in response to contralateral, ipsilateral and binaural stimuli were extracted. The example showed a contralateral preference where the binaural response clearly resembled the contralateral response. EPSCs and IPSCs that were induced by contralateral stimuli had higher current amplitudes (Figures 3C,E) and lower intensity thresholds (indicated by *gray dashed circles*, Figures 3A,B) than those induced by the ipsilateral stimuli. The thresholds of the ipsilateral and contralateral stimulation were 40 dB SPL and 10 dB SPL, respectively (same for the EPSC and IPSC; Figures 3A,B *gray dashed circles*). To quantify the relationship among the binaural, contralateral and ipsilateral responses, we plotted the contralateral synaptic amplitude against the binaural synaptic amplitude (Figures 3C,E *black line*) and the ipsilateral synaptic amplitude against the binaural synaptic amplitude (Figures 3C,E *red line*) to the same intensity (0–70 dB SPL) CF tone stimulus, as plotted for the same cell in Figure 3A. The binaural synaptic responses were linearly correlated with the contralateral responses, with a correlation coefficient (*r*) as high as 0.99 for both the excitatory (Figure 3C, *black line*, *r* = 0.99 and slope = 0.98) and inhibitory (Figure 3E, *black line*, *r* = 0.99 and slope = 0.97) currents. However, neither the excitatory (Figure 3C, *red line*, *r* = 0.89 and slope = 0.35) nor the inhibitory (Figure 3E, *red line*, *r* = 0.86 and slope = 0.53) binaural responses correlated with the ipsilateral responses.

For all recorded neurons, by plotting the correlation coefficient vs. the slope for both EPSCs (*n* = 23) and IPSCs (*n* = 25), we found a strong linear correlation between the levels of contralateral and binaural synaptic current responses with correlation coefficients close to 1 (Figures 3D,F *black clusters*). In contrast, the correlation between the ipsilateral and binaural synaptic current responses was much weaker (Figures 3D,F *red clusters*). Thus, the slope value could be considered for the evaluation of the relationship between the binaural response and contralateral input. A binaural synaptic response that was suppressed by ipsilateral input had a linear fit with a slope value >1. Meanwhile, a slope value < or = 1 indicated that the influence of the ipsilateral input was facilitatory or ineffective, respectively.

The thresholds of EPSCs (Figure 3G) and IPSCs (Figure 3I) from ipsilateral stimuli were higher than those from contralateral stimuli on most neurons. In addition, the threshold for ipsilateral stimuli was significantly higher than that for contralateral and binaural stimuli, but there was no difference between the binaural and contralateral intensity thresholds, both for EPSCs (Figure 3H; one-way ANOVA, *p* < 0.001; LSD tests for multiple comparisons, *p* = 0.598 (contra-binaural)) and IPSCs (Figure 3J; one-way ANOVA; *p* < 0.01, LSD tests for multiple comparisons, *p* = 0.556 (contra-binaural)).

It was found that contralateral inputs could be modified, including the unchanged, facilitated, or inhibited effects, by the ipsilateral stimulation.

### Monaural Selectivity of the Binaural Acoustic Response to an Intensity-Intensity Scan

**Figure 4:**
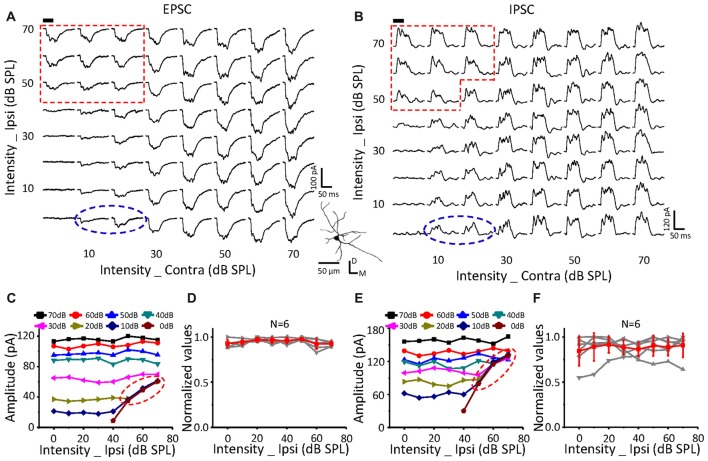
Monaural selectivity on the neurons with ipsilateral inputs having no effect on the contralateral response. **(A,B)** CF tone-evoked excitatory **(A)** and inhibitory **(B)** responses to the binaural stimulus array; CF = 12125 Hz. Each subplot shows the postsynaptic currents (average of 10 repeats). Tone bursts (50 ms duration) are indicated in the black bar. The right corner shows the reconstructed morphology of the recorded cells labeled with biocytin. The red region indicates the binaural selective region. **(C,E)** The peak amplitudes of excitatory **(C)** and inhibitory **(E)** currents in response to contralateral and ipsilateral stimulation at different intensities plotted for the cell shown in **(A,B)**. **(D,F)** The normalized excitatory **(D)** and inhibitory **(F)** response amplitude curves of ipsilateral (0–70 dB SPL) and contralateral (fixed at 50 dB SPL) inputs in all the recorded neurons.

**Figure 5:**
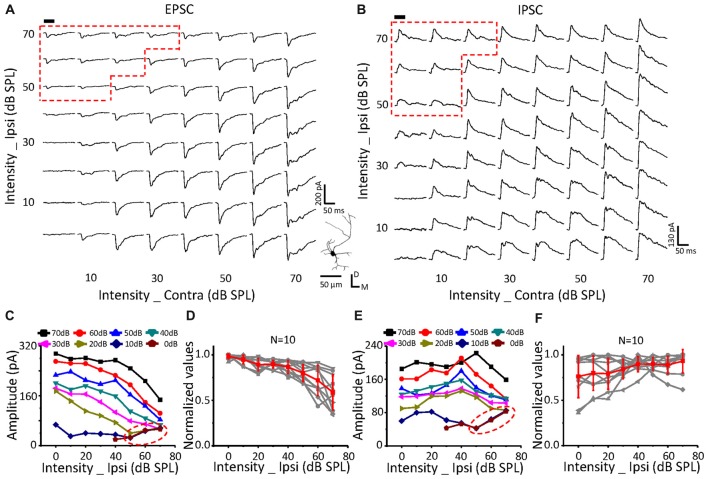
Monaural selectivity on the neurons with ipsilateral inputs inhibiting the contralateral response. **(A,B)** CF tone-evoked excitatory **(A)** and inhibitory **(B)** responses to the binaural stimulus array; CF = 14928 Hz. For details see Figures 4C–F.

**Figure 6:**
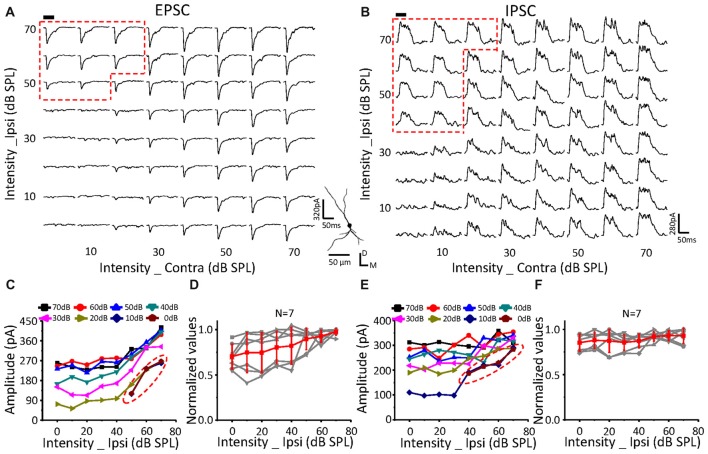
Monaural selectivity on the neurons with ipsilateral inputs facilitating the contralateral response. **(A,B)** CF tone-evoked excitatory **(A)** and inhibitory **(B)** responses to the binaural stimulus array; CF = 24251 Hz. For details see Figures 4C–F.

The effects of ipsilateral excitatory and inhibitory inputs on the corresponding binaural responses could be different on the same ICC neurons. Therefore, based on the EPSC slope value between the binaural and contralateral response (Figure 3D, *black clusters*), three types of neurons were categorized. A slope value =, > or, <1 indicated ineffective (Figure 4), suppressive (Figure 5), or facilitative (Figure 6) effects, respectively. To further examine the relationship of postsynaptic responses with monaural and binaural stimulations, we adopted an intensity-intensity scan in which the levels of the ipsilateral and contralateral stimuli varied from 0 dB SPL to 70 dB SPL.

On a neuron with no effects of ipsilateral inputs on the binaural acoustic response (Figures 4A,B) for both EPSC and IPSC, the thresholds of the ipsilateral and contralateral stimuli were 40 dB SPL and 10 dB SPL, respectively. A special response region was observed (Figures 4A,C: EPSC; Figures 4B,E: IPSC; *red dotted line*) in which the binaural responses were exactly the same as the ipsilateral synaptic inputs. This indicated that the ipsilateral inputs could be the only contributor, instead of being superimposed with the contralateral inputs (*dashed blue circles*), when ipsilateral inputs were large enough.

In total, six neurons were found in this study with no obvious changes in binaural excitatory and inhibitory synaptic responses when the contralateral stimuli were fixed at 50 dB SPL, and the intensity of the ipsilateral stimuli gradually increased (Figures 4D,F). In addition, special response regions similar to the example in Figures 4A,B (*red dotted line*) were found on all of them.

The same relationship was observed for neurons with suppressive (Figure 5, *n* = 10) or facilitative (Figure 6, *n* = 7) effects of ipsilateral inputs on the binaural acoustic response as observed in neurons with no ipsilateral-binaural effects (Figure 4). Although the binaural excitatory and inhibitory responses could be affected by the corresponding ipsilateral inputs in a different way on the same ICC neurons (Figures 5, 6D,F), the special response regions where only the ipsilateral inputs determined the binaural responses always existed (Figures 5, 6A,C EPSC; Figures 5, 6B,E IPSC;* red dotted line*).

In summary, regardless of how the ipsilateral inputs affected the contralateral inputs, the binaural acoustic-evoked synaptic responses were selective for the ipsilateral inputs or the modified contralateral inputs depending on their strength.

### A “Switch” Point to Evaluate the Monaural Selectivity

**Figure 7:**
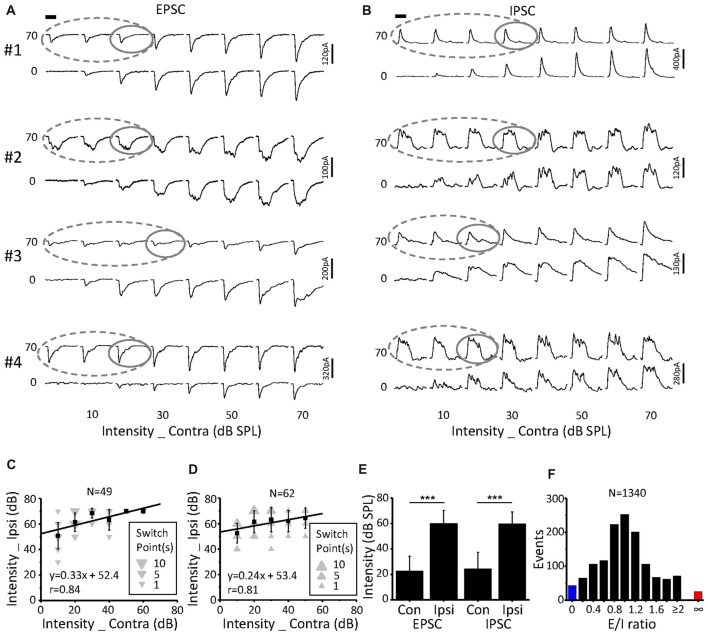
Characteristics of the switch points. **(A,B)** CF tone-evoked excitatory **(A)** and inhibitory **(B)** synaptic responses for four examples of ICC neurons. The gray solid line represents the switch point. Within this region, the binaural responses were the same as the ipsilateral responses (gray dashed lines). Tone bursts (50 ms duration) are indicated by the black bar. **(C,D)** The distribution of excitatory **(C)** and inhibitory **(D)** synaptic response switch points in the recorded population. Data are expressed as the mean ± SD. **(E)** Comparison of the average switch point intensity of EPSCs and IPSCs. ***p < 0.001, paired *t*-test. **(F)** Distribution of the excitation/inhibition ratio. The blue and red columns indicate the abnormal E/I ratio where there are only EPSCs or IPSCs, respectively.

Because the binaural synaptic response had monaural selectivity (Figures 4–6), we defined the cutoff between the contralateral and ipsilateral dominations as a switch point in this study. In detail, when the ipsilateral stimulus intensity was fixed, the contralateral stimulus intensity presented in a decreasing phase, at which the ipsilateral stimulus induced the same response (based on the peak amplitude, half-peak duration and the latency of synaptic response) as the binaural stimulus; this was termed the “switch” point. For example, after the ipsilateral stimulus intensity was set as 70 dB SPL (upper traces) or 0 dB SPL (lower traces, Figures 7A,B) the response amplitudes as a function of the contralateral stimulus intensity were extracted for both the EPSCs (Figure 7A) and IPSCs (Figure 7B). Within the region where the binaural responses were the same as the ipsilateral responses (*gray dashed lines*), the switch point was the one with the largest contralateral stimulus intensity (Figures 7A,B *gray solid lines*).

A total of 49 switch points were identified in 19 out of the 23 neurons for EPSCs. These switch points had linearly correlated ipsilateral and contralateral intensities (Figure 7C, *r* = 0.84, intercept = 52.4 dB SPL). In comparison, the switch points for IPSCs (*n* = 62) were found in 22 out of the 25 neurons. There was also a clear correlation between the ipsilateral and contralateral stimulus intensities (Figure 7D, *r* = 0.81, intercept = 53.4 dB SPL). For EPSCs, the ipsilateral intensities (60.2 ± 9.9 dB SPL) of the switch points were significantly higher than the contralateral intensities (22.7 ± 11.7 dB SPL, paired *t*-test, *p* < 0.001; Figure 7E). The same result was observed for the IPSCs (Figure 7E; ipsilateral, 59.8 ± 9.3 dB SPL; contralateral, 24.5 ± 12.8 dB SPL; paired *t*-test, *p* < 0.001).

Excitation-inhibition balance is a fundamental feature of sensory neuronal information processing. The distribution of the E/I ratio on the recorded neurons (*n* = 23, 8 × 8 stimulation array) is shown in Figure 7F. We found that both EPSCs and IPSCs were evoked for most stimulation events (Figure 7, 94.9%, *black*). However, only EPSCs (Figure 7F, 1.9%, *red*) or IPSCs (Figure 7F, 3.2%, *blue*) were observed when the corresponding stimulation was lower than the threshold to IPSC or EPSC, respectively. This result suggested that there is an imbalance of EPSCs and IPSCs near the threshold level, which is consistent with findings that were reported for the auditory cortex (Zhao et al., 2015).

## Discussion

In this study, we tested the ICC neuronal synaptic responses to the CF tone in anesthetized mice. We found that the binaural synaptic responses corresponded to the contralateral synaptic response modified by the ipsilateral stimuli. As the ipsilateral stimulus intensity increased, the binaural synaptic response showed significant shifts towards the ipsilateral synaptic response. When their intensities were larger than the “switch” point, no matter how the ipsilateral inputs affected the contralateral inputs, the ipsilateral inputs were the only contributors to the binaural synaptic response.

The IC includes three subregions, the ICC, the dorsal cortex of the ICD, and the external nucleus of the ICX. The ICC with a dorsal-to-ventral gradient of CF (from low to high) is an important ascending station for frequency processing (Stiebler and Ehret, 1985; Yu et al., 2005). The ICD and ICX neurons receive more complex information not only from the brainstem nuclei, such as the CN, superior olive complex (SOC) and NLL but also from the auditory cortex (Barnstedt et al., 2015; Xiong et al., 2015) and other nuclei (Wu et al., 2015). Since these three subregions are close to each other, the ICC region was previously identified based on short response latencies, the depth of the recording sites, and sharply tuned TRFs (Willott, 1984; Yu et al., 2005). We also used these criteria to roughly identify the ICC neurons during the electrophysiological recordings. Moreover, we labeled the recorded neurons with biocytin to identify their accurate locations (Figure 1E) after recording. Only data obtained from the neurons located in the ICC were further analyzed in this study.

The ICC neurons receive ipsilateral and contralateral (Glendenning et al., 1992; Oliver et al., 2003; Loftus et al., 2004; Greene et al., 2010) projections from lower brainstem nuclei. There has been some controversy over whether binaural information is integrated in the brainstem or within the ICC. If the contralateral and ipsilateral inputs were integrated within the ICC, the binaural excitatory or inhibitory responses should be the summation of the corresponding contralateral and ipsilateral inputs or should be, at least, larger in amplitude than either of them. However, the binaurally evoked excitatory current was much smaller than the summation of the ipsilaterally and contralaterally evoked excitatory currents (Figures 4–6, Xiong et al., 2013). In addition, the binaural excitatory currents were exactly the same as the currents evoked by ipsilateral stimuli when the ipsilateral responses were significantly higher than the contralateral responses (Figures 4–6C). Therefore, there is no obvious integration in the ICC, and binaural information integration should be performed in the brainstem.

The ICC receives excitatory inputs from the ipsilateral medial superior olive (MSO) and contralateral LSO, which are the important nuclei underlying the binaural information integration (Adams, 1979; Glendenning et al., 1981; Grothe et al., 2010). Most MSO neurons belong to binaurally excitable (EE) neurons (Goldberg and Brown, 1968; Yin and Chan, 1990). Meanwhile, most LSO neurons have EI properties (Caird and Klinke, 1983). Therefore, the ICC neurons being facilitated by ipsilateral inputs likely inherited the integrated information from the MSO, while the neurons being inhibited by ipsilateral inputs likely inherited information from the LSO. For neurons with no-modified effects of ipsilateral inputs on the binaural responses, more complicated integrations of binaural information might exist. Further investigations are needed to confirm the sources of synaptic inputs received by ICC neurons. Thus far, although we were unsure about the exact nuclei where the integrations take place, the “switch” point was found in the majority of the neurons recorded in this study (19 out of the 23 neurons, Figure 7C). We could not completely exclude the probability that the binaural information could also be integrated within the ICC in the neurons without an obvious “switch” point (*n* = 4). As with the excitatory responses, the inhibitory responses had similar performances (Figures 4–6D), and switch points were also found (Figure 7D). Because the ICC receives inhibitory inputs from the bilateral DNLL, as well as from the LSO on the same side (Brunso-Bechtold et al., 1981; Saint Marie and Baker, 1990; Glendenning et al., 1992; Moore et al., 1998; Casseday et al., 2002), we speculate that the inhibitory inputs may be integrated in these nuclei before they reach the ICC neurons.

Additionally, different performances of the EPSCs and IPSCs were found that concern the selectivity of the ipsilateral or contralateral stimulation (Figures 4–6A,B indicated by the shapes of the dashed red lines). This might be because the brainstem nuclei that integrate and transfer the excitatory and inhibitory inputs to the ICC neurons were different. Meanwhile, this difference in the excitatory and inhibitory input performances would induce the complex outputs in the ICC neurons. This suggested that the ICC neurons could further process the binaural acoustic information, although they inherited the integrated information from the lower nuclei.

Balanced excitatory and inhibitory synaptic inputs were found in response to the ILD that varied around a constant average binaural level (ABL) in the IC (Ono and Oliver, 2014). Our results obtained by performing an intensity-intensity scan were in agreement with Ono and Oliver (2014) view that EPSCs and IPSCs varied consistently with ILDs in ABL (Figures 4–6). However, different changing patterns of EPSCs (Figure 5D) and IPSCs (Figure 5F) were found in our present study by comparing them on the same cells for contralateral-level (CL)-constant stimulation. In Figure 5C, for example, when the contralateral stimulus levels were fixed at 50 dB SPL, the peak amplitude of the binaural EPSC was suppressed as the ipsilateral intensity increased. The IPSCs, however, had nonmonotonic responses, as shown in Figure 5E. This suggested that the excitatory and inhibitory synaptic inputs changed in a complex way as a function of binaural stimulation. The contributions of the ipsilateral and contralateral inputs to the binaural acoustic-evoked synaptic responses on ICC neurons should be helpful for further understanding the underlying synaptic mechanism of sound localization.

## Author Contributions

ZX conceived and designed the study. JW, WZ, CX, YL and CS performed the research. JW, WZ, CX, YL and CS analyzed the data. ZX wrote the article.

## Conflict of Interest Statement

The authors declare that the research was conducted in the absence of any commercial or financial relationships that could be construed as a potential conflict of interest. The reviewer TB and handling Editor declared their shared affiliation, at the time of the review.
